# Total diet, individual meals, and their association with gastroesophageal reflux disease

**DOI:** 10.15171/hpp.2017.28

**Published:** 2017-06-14

**Authors:** Mehranghiz Ebrahimi-Mameghani, Siamak Sabour, Manouchehr Khoshbaten, Seyed Rafi Arefhosseini, Maryam Saghafi-Asl

**Affiliations:** ^1^Nutrition Research Center, Department of Nutrition in Community, School of Nutrition & Food Sciences, Tabriz University of Medical Sciences, Tabriz, Iran; ^2^Safety Promotion and Injury Prevention Research Center, Department of Clinical Epidemiology, School of Health, Shahid Beheshti University of Medical Sciences, Tehran, Iran; ^3^Professor in Gastroenterology and Hepatology, Drug Applied Research Center, Tabriz University of Medical Sciences, Tabriz, Iran; ^4^Nutrition Research Center, Department of Biochemistry & Diet Therapy, School of Nutrition & Food Sciences, Tabriz University of Medical Sciences, Tabriz, Iran

**Keywords:** Gastroesophageal reflux disease, Diet, Fat, Meal volume, Caloric density

## Abstract

**Background: ** To identify the association of total diet and individual meals with gastroesophageal reflux disease (GERD).

** Methods:** This age- and sex-matched case-control study was carried out among 217 subjects (106 cases and 111 controls). Data were collected using a demographic questionnaire and a GERD checklist and a 3-day food record.

**Results:** Cases consumed more fat (median: 26.3 [3.2-71.5] g vs. 21.8 [4.3-58.1] g; P=0.04)and more energy percent form carbohydrates (median: 72.5 [0-100] vs. 69.0 [0-100]; P=0.02)at lunch, and less energy (median: 129.5 kcal [0-617.6] vs. 170.5 kcal [0-615.7]; P=0.01) and protein (2.4 [0-19.4] g vs. 3.1 [0-21.8] g; P=0.01) at evening snack, compared to controls.The volume of food was significantly different between the two group only at lunch (median:516 [161-1292] g vs. 468 [198-1060] g; P=0.02). The percentage of energy from total dietary protein showed a significant association with GERD after adjusting for confounders (odds ratio[OR]=0.89; 95% CI: 0.81-0.98). Regarding the individual meals, amount of fat consumed at lunch (OR=1.02; 95% CI: 1.00-1.05), and amount of protein intake at evening snack (OR=0.92;95% CI: 0.85-1.00) were significantly associated with GERD. Meanwhile, caloric density and meal frequency did not differ significantly between the two groups.

**Conclusion:** Amount of fat consumed at lunch is positively associated with GERD, whereas the percentage of energy from total protein and amount of protein intake at evening snack are more likely to be inversely associated with GERD.

## Introduction

Gastroesophageal reflux disease (GERD (is a health problem with a high prevalence and remarkable consequences such as esophageal stricture, gastrointestinal bleeding, or Barrett’s esophagus for those affected.^1^ It is a pathologic condition of the esophagus caused by regurgitation of gastric- or gastroduodenal contents into the lumen of the esophagus.^2^ Epidemiologic studies showed that the prevalence of GERD is between 10% to 48% in western countries and up to 5% in Asia^3^; however, an increasing trend is reported.^4^ In Iran, according to a population-based study,^5^ it is estimated to be up to 33% among adults. The typical symptoms of reflux are heartburn (a retrosternal burning sensation) and acid regurgitation (a sour taste in the mouth).^6^

The symptoms ofGERD can trigger esophagitis to such an extent as to lessen the patient’s quality of life.^7^ Furthermore, GERD is a strong risk factor for Barrett’s esophagus^8^ and esophageal adenocarcinoma (EA).^9^ A systematic review on GERD indicated both absence from work and reduced productivity while at work.^10^ As the incidence of EA has increased in the world over the last 30 years,^11^ it is very important to identify factors which may affect the conditions leading to the development of GERD.

Despite the importance of GERD and remarkable knowledge of its pathogenesis,^12^ risk factors remain poorly understood. However, there is considerable evidence that GERD occurs more commonly after meals.^13,14^ In this regard, majority of studies have been carried out from physiological,^15,16^ but not dietary perspective. They have investigated the effect of fat on postprandial esophageal acid exposure,^17,18^ or on lower esophageal sphincter (LES) motility.^19,20^ In addition, they were mostly in patients with severe GERD and even hospitalized patients with esophagitis and EA.^21,22^

Most of the physiological studies failed to establish the role of diet (most notably fat) on GERD or altering the competence of LES junction.^23,24^ Even some suggested that recommending a low-fat diet to GERD patients is, however, an inappropriate approach.^20,24^ In contrary, some of the prior investigators demonstrated the association of fat with GERD.^21,25^ Therefore, relevant studies have often provided conflicting results.^26,27^

Generally, studies investigating the effect of total dietary intake on GERD are scarce.^28,29^ Amongst is a cross-sectional study by El-Serag et al^28^ in 371 employees at Veteran Administration in which a significant association was reported between high fat intake and GERD symptoms; however, having adjusted the effect of body mass index (BMI), the association became non-significant; since there is a significant association between obesity and GERD symptoms.^30^ A more recent study suggested that further work investigating the association between dietary fat intake and food sources of fat are needed for conﬁrmation of these results.^31^

Present physiological studies on meal volume and caloric density in GERD are inconclusive. Iwakiri et al^32^ reported a decrease in postprandial GERD by reducing the volume of a liquid meal in healthy volunteers. Pehl et al^33^ showed that the amount of gastroesophageal reflux induced by ingestion of a meal seems to depend on the volume but not on the caloric density of a meal. In contrast, the study by Colombo et al^24^ indicated that advice on dietary habits in GERD patients should be concentrated on decreasing the caloric load of meals rather than their fat content. However, Esmaillzadeh et al^34^ reported certain associations between dietary patterns and GERD, which may partly be modulated by body weight.

Health promotion of the population particularly those with GERD is important and to the best of our knowledge, the role of individual meals of diet on GERD has not been studied yet. Therefore, in the present study the association of total diet as well as individual meals was investigated to bridge the gap in this area.

## Materials and Methods

### Participants

A total of 250 subjects consented to participate in our case-control study. Of these, 217 continued the study. The sample frame was selected among patients referred to the specialized clinic of Tabriz University of Medical Sciences for different health-seeking purposes. This clinic is the major provider of medical care in Tabriz, northwest of Iran. Enrolled subjects were all 14 years or older and were requested to complete an informed consent.

### Study protocol

Subjects were asked whether they had experienced recurrent heartburn, acid regurgitation, or both at least monthly during the prior 12 months; if so, they were referred to an experienced gastroenterologist for further investigation. Having confirmed the diagnosis of GERD, the subject was assigned to the case group. Age- and sex-matched subjects who did not experience any of the aforementioned symptoms over the past 12 months were selected as controls.

Since heartburn and acid regurgitation are the two main symptoms of GERD, assessing these symptoms could be reliable to measure the true occurrence of reflux and to allow appropriate treatment.^35,36^ Therefore, these two symptoms are considered specific for GERD.^37^ They can be used to make the diagnosis of GERD without additional tests.^38^

Therefore, GERD diagnosis was based on a GERD symptom checklist. This included specific questions about the type and frequency (at least weekly or monthly) of symptoms. Endoscopy, being invasive, was offered only to suspected patients, if they had consent.

The following exclusion criteria were applied at baseline: gastric surgery, esophageal or gastric cancer, history of vagotomy, confirmed peptic ulcer disease, dieting such as weight loss diet, use of LES-motility changing drugs such as calcium-channel blockers and nitrates, proton pump inhibitors (PPIs), H_2_ receptors antagonists (H_2_-RA), and contraceptive/hormonal medications. Antacid medication, if positive, was stopped one month prior to food record.

### Measurements

Data including age, marital status, education level, occupation, smoking and post-menopausal status were also gathered and BMI was calculated as weight (kg)/ height (M^2^) according to Quetelet’s formula.^39^ Furthermore, the interviewed subjects were sent home with a 3-day food record diary in order to provide more accurate and reliable estimation of food intake. To meet this demand, type and amount of dietary macronutrient components, consumed on two weekdays and one weekend in each meal were questioned. Subjects were instructed by a trained dietitian to consume their usual diet. Meanwhile, they were trained on how to fill out the diary. Upon the form completion, an in-person interview was conducted with subjects so as to be ensured that those foods recorded were typical of their routine diet. Then, the data on total diet as well as each meal were analyzed using Nutritionist III software (Axxya Systems, Stafford, TX), modified for Iranian foods. Caloric density of foods was calculated as the available energy per unit weight of food (kcal/g) excluding non-caloric beverages and drinking water. The sample size was estimated, based on mean fat intake with 80% power and α-error of 5% and a case to control ratio of 1:1, using literature-derived data^40^; the effect size for dietary fat was 2.4 g (standard deviation [SD] = 6). It was predicted that 99 persons in each group would detect changes in serum dietary parameters, using the two-means formula. However, we recruited 217 persons (106 cases and 111 controls) for the study.

### Statistical analysis

Statistical analysis of the data was carried out using SPSS version 16 for Windows (PASW Statistics; SPSS Inc., Chicago, IL, USA). For all continuous variables, median and range were presented and X^2^ was performed to test associations of categorical variables. Mann-Whitney U test and student *t* test were used for comparing means of variances between the two groups. The association of GERD with specific risk factors was reported as odds ratio (OR) and 95% CI, using a logistic regression model that “no reflux” was the reference category. Univariate logistic regression was performed to evaluate the association between GERD and dietary items. Furthermore, the associations were assessed based on fitting multiple logistic regression models adjusted for BMI and education level. All calculated *P* values were two-sided and *P* values less than 0.05 were considered statistically significant.

## Results

Out of 250 participants who filled out the GERD checklist, 217 returned their dietary records with complete and interpretable answers, among whom 106 (48.8%) had experienced GERD symptoms (case group) with a mean age (±SD) of 35.3 ± 12.6 years and 111 (51.2%) were controls with a mean age of 35.1 ± 13.2 years. Only education level differed significantly between cases and controls (*P* < 0.001; Table 1).

Among 106 cases, 69 (65.1%) had at least weekly symptoms while 37 (34.9%) complained of monthly symptoms. Total diet and individual meals of the participants are summarized in Table 2. Cases and controls showed statistically non-significant differences. Even though cases reported further total energy intake when compared to controls (median, 1922.5 kcal vs. 1882 kcal), however, their difference did not reach a significant level. Both groups consumed similar amounts of carbohydrates (median, 265.7 g vs. 272.1 g), protein (median, 60.6 g vs. 63.7 g), and fat (median, 62.4 g vs. 61.4 g). Similarly, the intake of other nutrients was more or less the same. Meanwhile, total caloric density, total meal volume and frequency did not differ significantly between the two groups (Table 3). In contrast, statistically significant differences were observed between participants with and without GERD at lunch and evening snack for the following dietary items; at lunch: amount of fat (*P* = 0.04) and meal volume (*P* = 0.02), and at evening snack: energy intake (*P* = 0.01), amount of protein (*P* = 0.01), fat (*P* = 0.01), and sugar (*P* = 0.03), and percentage of energy from carbohydrates (*P* = 0.02). Amount of carbohydrates and caloric density at evening snack were marginally significant (*P* = 0.05).

Results of multivariate logistic regression revealed significant associations between GERD and percentage of energy from protein (OR = 0.89; 95% CI: 0.81-0.98) in total diet; amount of fat (OR = 1.02; 95% CI:1.00-1.05) at lunch; amount of sugar (OR = 0.95; 95% CI:0.91-0.99) and energy intake (OR = 0.99; 95% CI:0.99-1.00) at evening snack with GERD after adjusting for BMI and education level (Table 4).

## Discussion

Because GERD is an important health problem and modification of dietary behavior appears to play a role in its prevention, we studied the association of total diet as well as individual meals (including large meals and snacks) with GERD. The results showed that among total dietary factors, only percentage of energy from total protein was significantly associated with GERD.

To the best of our knowledge, this is a preliminary study that examined the association of total diet as well as individual meals (including large meals and snacks) with GERD. Our study demonstrated that total dietary intakes of cases and controls did not differ significantly. In addition, total dietary factors had no significant association with GERD, except for percentage of energy from total protein. It could be in part explained by the fact that protein increases the LES pressure and stimulates gastrin secretion which promotes stomach emptying.^41,42^ Prior investigators have postulated a pathophysiologic relationship between delayed gastric emptying,^43^ decreased LES pressure^25^ and GERD.

El-Serag et al^28^ reported significantly higher daily intakes of total fat, saturated fatty acids, percentage of energy from fat, and average fat servings in GERD patients comparing with healthy subjects. Moreover, there was a dose-response relationship between GERD and fatty acids and cholesterol. Though after adjusting for BMI, the association between fat and GERD was non-significant, however, in their study food intake was only evaluated by a food frequency questionnaire which is prone to recall bias, whereas in our study a 3-day food record was obtained, the gold standard tool of dietary assessment.^44^

In another study,^33^ performed on 60 patients with reflux, perceived reflux event was significantly associated with higher intakes of cholesterol, saturated fatty acids, and calories from fat.This study demonstrated that BMI did not correlate with having a sensed reflux event. However, their study suffered not only from a small number of patients, but also it lacked control group. In contrary, our study had adequate power to detect the differences between the two groups and had control group as well. In a study by Nandurkar et al*,*^29^ no significant association was found between diet and reflux symptoms in 211 community subjects.

In our study, there was a great variability in reported amount of foods consumed particularly at meals in both groups (Table 2). In addition, it is unknown whether GERD patients had altered their diet at the time of the study, since GERD patients are often advised to adjust their dietary habit, e.g. by minimizing the intake of high-fat meals or excluding offending foods to avoid symptoms. Nonetheless, some of the patients with non-severe GERD were reluctant to change their diet despite persisting symptoms; perhaps, because of the pleasure of eating those foods. Besides, it is not clear whether patients were exposed to symptoms at the time of food record, or it influenced the type and amount of the food consumed. The above mentioned factors may interfere with significant associations between total diet and GERD.

Although it was revealed that there is almost no significant association between total dietary intake and GERD, several food items consumed at certain meals were found to be associated. For example, as expected, a significant association was found at lunch for amount of fat and marginally for meal volume. Lunch comprised the largest and main meal among most individuals. In addition, an enhanced volume might increase GERD via an enhanced gastric distension; thereby, triggering transient LES relaxations (TLESRs), considered the predominant mechanisms of reflux events in healthy subjects and reflux patients.^33^ On the other hand, extracellular fats are widely used at lunch. Therefore, fat consumption at lunch might contribute to postprandial GERD symptoms.

Unlike lunch, it appears that evening snack is more likely to show a protective effect of energy, protein, fat, and sugar on GERD, regardless of the type and the food consumed. More interestingly, fat consumed at lunch provokes GERD symptoms; whilst at evening snack alleviates the symptoms. However, the mechanism (s) for such an effect is unclear; but as fat is consumed in different forms (e.g. intracellular vs. extracellular fat) and in varying proportions with other macronutrients, hence, the physiologic response to fat ingestion may potentially vary from one meal/snack to another.^45^ In our study, fat was used in extracellular form at lunch, while it was ingested in intracellular form at evening snack, i.e. in the form of sweets, cakes, biscuits and junk foods. Therefore, the form of the consumed fat (e.g. intracellular vs. extracellular fat) might explain the present finding.

Given that sweets have been regarded as causing reflux because of their high osmolality and high fat content, it is expected to have more links with GERD at evening snack. However, it seems to be unlikely at least in our study; since this snack had minor volume of fat, sugar, carbohydrates, and so on, especially in GERD patients (Table 4); that is why the effect is more likely to be reversed. Although snacking may promote energy imbalance resulting in obesity among different age groups,^46^ our results suggest evening snacking might protect GERD patients against the disease.

### Limitations 

We acknowledge some limitations. This is a clinical-based, but not a population-based study. However, as mentioned above, we used the clinic of Tabriz Medical University which is a major provider of medical care in Tabriz, northwest of Iran. In addition, misclassification might exist since endoscopy was not offered to all patients, due to low compliance, however, suspected cases were not included and only those who had at least monthly symptoms of GERD were recruited. Finally, we could not investigate the association between diet and reflux severity.

## Conclusion

In conclusion, to promote health among population, particularly patients with GERD, dietary modification through following dietary guidelines and meal management and having appropriate food choices in each meal play an important role in the management of GERD. Our results indicate the protective effect of the percentage of energy from protein in total diet. It also shows that lunch and evening snack are associated with GERD symptoms. Seemingly, amount of fat at lunch positively affects GERD, whereas amount of macronutrients at evening snack reduces the symptoms, provided that the meal volume be low. Therefore, recommending a low-fat meal, particularly at lunch, to GERD patients sound reasonable. This is clinically important and may be suggested in the guideline for management of GERD. The increasing prevalence of patients with GERD along with inappropriate dietary habits merits evaluation of a proper dietary intervention for GERD and its symptoms. Large-scale studies are required to evaluate the impact of total diet as well as individual meals on symptoms.

## The recommendation for practical implications and policy making

Regarding the important effect of diet in the management of GERD, it seems sound to accentuate more on dietary items in the current guidelines of GERD.

## Ethical approval

The Ethics Committee of Tabriz University of Medical Sciences approved the protocol for the study (Reference Number: 5/4/11127).

## Competing interests

The authors report no competing interests.

## Authors’ contribution

EM M contributed in the study design, data interpretation, and appraised the manuscript. S S helped with data analysis and commented on the draft of the manuscript. AH SR contributed in the study design and appraised the manuscript. K M helped with the study design, acquisition of data, and reviewing the draft of the manuscript. SA M participated in the study design, acquisition of data, questionnaire development, data analysis and interpretation, drafting and editing the manuscript. All authors approved the final version for submission.

## Funding

The present study was funded by Research Vice-Chancellor, Tabriz University of Medical Sciences, Tabriz, Iran.

## Acknowledgements

We acknowledge all the participants as well as the staff of the specialized clinic of Tabriz University of Medical Sciences for their kind cooperation. 

**Table 1:** Socio-demographic characteristics in participants with and without GERD symptoms

**Variable**	**GERD symptoms** **(n=106), n (%)**	**No GERD symptoms** ** (n=111), n (%)**	***P*** ^a^
Age (y), Mean ± SD	35.3±12.6	35.1±13.2	0.91
Gender			
Male	27 (25.4)	30 (27.1)	0.42
Female	79 (74.6)	81 (72.9)	
Occupation			
Unemployed	89 (84.0)	91 (82.0)	0.11
Non-governmental	10 (9.4)	13 (11.7)	
Retired	3 (2.8)	8 (7.2)	
Housewife	62 (58.5)	46 (41.4)	
Student	14 (13.2)	24 (21.6)	
Employed	17 (16.0)	20 (18.0)	
Education			
Illiterate	20 (18.8)	8 (7.2)	**0.007**
Literate	67 (63.2)	64 (57.6)	
Higher education	19 (17.9)	39 (35.1)	
Marital status			
Single	25 (23.5)	34 (30.6)	0.42
Married	81 (76.4)	77 (69.3)	
Smoking			
None-smoker	103 (47.5)	110 (50.7)	0.23
Menopausal status			
Yes	14 (17.9)	9 (11.4)	0.18
No	64 (82.1)	70 (88.6)	

Abbreviation: GERD, gastroesophageal reflux disease. ^a^Chi-square test.

**Table 2:** Comparison of total diet and main meals^a^ in participants with and without GERD symptoms

**Variables**	**GERD symptoms** ** (n=106)** **Median (range)**	**No GERD symptoms** ** (n=111)** **Median (range)**	***P*** ^b^
Total dietary intake			
Energy (kcal/d)	1922.5 (823-3815)	1882 (915.8-3698.7)	0.83
% Energy from carbohydrates	56.0 (35-77)	57.0 (42-72)	0.92
% Energy from protein	13.0 (8-25)	13.0 (8-25)	0.06
% Energy from fat	32.0 (11-53)	30.0 (16-48)	0.33
Carbohydrates (g/d)	265.7 (97.8-526.2)	272.1 (122.5-660.3)	0.68
Sugar (g/d)	21.1 (1.6-59.1)	23.0 (1.2-117)	0.30
Protein (g/d)	60.6 (28.2-125.1)	63.7 (33-151.7)	0.11
Fat (g/d)	62.4 (18.3-146.8)	61.4 (26.4-168.8)	0.78
Cholesterol (mg/d)	187.2 (8.8-733.5)	211.5 (25.4-598)	0.76
Breakfast			
Energy (kcal/d)	415.7 (50.1-1057)	355.7 (21.3-1323)	0.25
% Energy from carbohydrates	62.5(15-83)	60.0 (9-100)	0.12
% Energy from protein	12.0 (2-23)	12.0 (0-29)	0.63
% Energy from fat	25.0 (4-80)	28.0 (0-76)	0.23
Carbohydrates (g/d)	67.4 (5.7-202.2)	57.0 (5.3-186.0)	0.19
Sugar (g/d)	0 (0-11.0)	0 (0-12.1)	0.84
Protein (g/d)	11.9 (1.1-40.1)	11.4 (0-73.0)	0.52
Fat (g/d)	10.4 (1.3-40.5)	11.3 (0-59.4)	0.51
Cholesterol (mg/d)	36.4 (0-326.6)	28.6 (0-399)	0.91
Lunch			
Energy (kcal/d)	651.7 (158.5-1528)	589.2 (290.7-2744)	0.23
% Energy from carbohydrates	49.0 (23-81)	50.0 (29-82)	0.42
% Energy from protein	13.0 (6-31)	14.0 (6-29)	0.18
% Energy from fat	36 (5-63)	35.0 (8-59)	0.15
Carbohydrates (g/d)	76.7 (21.2-271.6)	69.1 (30.5-544)	0.44
Sugar (g/d)	2.8 (0-12.7)	2.7 (0-21.7)	0.13
Protein (g/d)	19.5 (7.0-62.7)	21.6 (6.5-96.6)	0.52
Fat (g/d)	26.3 (3.2-71.5)	21.8 (4.3-58.1)	**0.04**
Cholesterol (mg/d)	43.3 (0-286.1)	45.5 (0-354)	0.51
Dinner			
Energy (kcal/d)	440 (21.9-1146)	484 (0-1437)	0.49
% Energy from carbohydrates	51 (17-83)	54 (0-77)	0.46
% Energy from protein	15 (7-43)	17 (0-38)	0.42
% Energy from fat	31 (4-76)	29 (0-79)	0.15
Carbohydrates (g/d)	54.4 (4.6-184)	59 (0-288.4)	0.12
Sugar (g/d)	2.4 (0-19)	2.5 (0-113.2)	0.61
Protein (g/d)	16.8 (0.7-68.7)	17.9 (0-103.5)	0.36
Fat (g/d)	14.6 (0.08-78.2)	15.2 (0-71.6)	0.92
Cholesterol (mg/d)	45.7 (0-437.8)	65.3 (0-240)	0.24

Abbreviation: GERD, gastroesophageal reflux disease. ^a^Measured by a 3-day food record. ^b^ Mann-Whitney U test.

**Table 3:** Comparison of daily individual snacks^a^ between participants with and without GERD symptoms

**Variables **	**GERD symptoms** ** (n=106)** **Median (range)**	**No GERD symptoms** ** (n=111)** **Median (range)**	***P*** ^b^
Morning snack			
Energy (kcal/d)	86.6 (0-508.6)	95.0 (0-668)	0.65
% Energy from carbohydrates	68.0 (0-100)	70 (0-100)	0.72
% Energy from protein	7.0 (0-27)	6.0 (0-29)	0.93
% Energy from fat	18.0 (0-74)	18.0 (0-73)	0.58
Carbohydrates (g/d)	15.3 (0-72.4)	15.4 (0-87.6)	0.61
Sugar (g/d)	1.7 (0-19.8)	0 (0-28)	0.83
Protein (g/d)	1.6 (0-17.6)	1.8 (0-19.2)	0.62
Fat (g/d)	1.9 (0-20.6)	1.9 (0-31.4)	0.78
Cholesterol (mg/d)	0 (0-227)	0.5 (0-401)	0.23
Evening snack			
Energy (kcal/d)	129.5 (0-617.6)	170.5 (0-615.7)	**0.01**
% Energy from carbohydrates	72.5 (0-100)	69.0 (0-100)	**0.02**
% Energy from protein	7.0 (0-21)	7.0 (0-34)	0.10
% Energy from fat	17.5 (0-47)	21.0 (0-60)	0.13
Carbohydrates (g/d)	25.0 (0-119.5)	29.6 (0-104)	0.05
Sugar (g/d)	4.0 (0-28.8)	4.9 (0-39.3)	**0.03**
Protein (g/d)	2.4 (0-19.4)	3.1 (0-21.8)	**0.01**
Fat (g/d)	2.5 (0-28.7)	3.7 (0-40)	**0.01**
Cholesterol (mg/d)	0.1 (0-253)	0.6 (0-114.5)	0.40
Before-bed snack			
Energy (kcal/d)	97.1 (0-727.3)	84.7 (0-782)	0.12
% Energy from carbohydrates	74.5 (0-100)	73 (0-100)	0.71
% Energy from protein	7 (0-27)	7 (0-27)	0.57
% Energy from fat	13.5 (0-76)	11 (0-55)	0.54
Carbohydrates (g/d)	18.2 (0-139.3)	15.9 (0-75.3)	0.22
Sugar (g/d)	4.7 (0-45.1)	3.9 (0-24.6)	0.68
Protein (g/d)	1.7 (0-14)	1.5 (0-23.8)	0.23
Fat (g/d)	1.6 (0-20.1)	1.1 (0-48.1)	0.12
Cholesterol (mg/d)	0 (0-180.3)	0 (0-109.5)	0.16

Abbreviation: GERD, gastroesophageal reflux disease. ^a^Measured by a 3-day food record. ^b^ Mann-Whitney U test.

**Table 4:** Comparison of meal volume and caloric density of total diet and individual meals^a^ in participants with and without GERD symptoms

** Variables**	**GERD symptoms** **(n=106)** **Median (range)**	**No GERD symptoms** **(n=111)** **Median (range)**	***P*** ^b^
Volume of total diet (g)	1973.5 (818.7-3898)	1937 (724-3563)	0.25
Volume of individual meals (g) at:			
Breakfast	403 (13.6-1257)	388 (52-1088)	0.31
Morning snack	143 (0-512)	168 (0-811)	0.66
Lunch	516 (161-1292)	468 (198-1060)	**0.02**
Evening snack	278 (0-831)	310 (0-727)	0.49
Dinner	353.5 (26.2-956)	365 (0-894)	0.79
Before -bed snack	184 (0-797)	153 (0-924)	0.10
Caloric density of total diet (cal/g)	0.94 (0.49-1.74)	0.97 (0.54-3.02)	0.27
Caloric density of individual meals (cal/g) at:			
Breakfast	1.01 (0.34-4.50)	0.99 (0.14-4.67)	0.84
Morning snack	0.62 (0-5.98)	0.50 (0-5.84)	0.91
Lunch	1.27 (0.49-2.70)	1.34 (0.54-11.29)	0.27
Evening snack	0.49 (0-3.82)	0.56 (0-3.73)	0.05
Dinner	1.36 (0.3-2.70)	1.33 (0-3.31)	0.89
Before- bed snack	0.46 (0-5.95)	0.42 (0-5.25)	0.24
Meal frequency	6 (4-6)	6 (3-6)	0.37

Abbreviation: GERD, gastroesophageal reflux disease. ^a^Measured by a 3-day food record. ^b^ Mann-Whitney U test.
